# Comparison of Long-Term Outcomes between Peritoneal Dialysis Patients with Diabetes as a Primary Renal Disease or as a Comorbid Condition

**DOI:** 10.1371/journal.pone.0126549

**Published:** 2015-05-11

**Authors:** Yutian Lei, Yifan Xiong, Lin Zhang, Hao Yan, Zhenyuan Li, Liou Cao, Jiaying Huang, Aiping Gu, Zhaohui Ni, Jiaqi Qian, Wei Fang

**Affiliations:** Department of Nephrology, Ren Ji Hospital, School of Medicine, Shanghai Jiao Tong University, Shanghai Center for Peritoneal Dialysis Research, Shanghai, China; University of Utah School of Medicine, UNITED STATES

## Abstract

**Objective:**

To investigate the long-term outcomes of peritoneal dialysis (PD) patients with diabetes as primary renal disease and patients with diabetes as a comorbid condition.

**Methods:**

All diabetic patients who commenced PD between January 1, 1995 and June 30, 2012 at Ren Ji Hospital, China were included. Patients were divided into diabetic nephropathy group (DN group) and non-diabetic nephropathy group (NDN group) according to their diagnosis of primary renal disease at the initiation of PD. They were followed until death, cessation of PD, transferred to other centers or to the end of study (June 30, 2013). Outcomes were analyzed by Kaplan-Meier method and Cox regression models.

**Results:**

A total of 163 diabetic patients were enrolled in the study, including 121 (74.2%) in DN group and 42 (25.8%) in NDN group. The 1-, 2-, 3- and 5-year patient survival rates were 89%, 78%, 66% and 51% for DN group, and 85%, 63%, 53% and 25% for NDN group, respectively. Kaplan-Meier analysis showed that patients in NDN group had a worse patient survival compared with DN group (log rank 4.830, P=0.028). Patients in NDN group had a marginally shorter peritonitis-free period (log rank 3.297, P=0.069), however, there was no significant difference in technique survival (log rank 0.040, P=0.841). Multivariate Cox regression analysis showed that older age (HR 1.047, 95% CI 1.022-1.073, p<0.001), cardiovascular disease comorbidity (HR 2.200, 95% CI 0.1.269-3.814, P=0.005) and diabetes as a comorbidity condition (HR 1.806, 95% CI 1.003-3.158, P=0.038) were the independent predictors of increased mortality.

**Conclusions:**

PD patients with diabetes as a comorbidity had an inferior patient survival compared to those with diabetic nephropathy, and closer monitoring and extra attention in the former subgroup of patients are therefore warranted.

## Introduction

Diabetes mellitus (DM) has become the most prevalent cause of end-stage renal disease (ESRD) in many western countries. Although the mortality rate for diabetic patients on peritoneal dialysis (PD) has improved considerably, it still remains inferior compared to their non-diabetic counterparts [1, 2].

However the issue of diabetes among ESRD was almost always raised together with diabetic nephropathy, while the number of patients with diabetes as a comorbidity was substantial [3]. It was hypothesized that patients with diabetes as a comorbidity may have a relatively lower burden of microvascular and macrovascular complications and hence a better outcome [4, 5]. However, there were few studies focusing directly on this issue and the results on the survival were variable [4–6]. This controversial question is still not fully understood and few available studies have attempted to delineate the long-term outcomes of these two groups of diabetic PD patients. Therefore we conducted this retrospective study to investigate the long-term outcomes of PD patients with diabetes as primary renal disease and those with diabetes as a comorbid condition.

## Materials and Methods

### Ethics Statement

The ethics committee of Ren Ji Hospital, School of Medicine, Shanghai Jiao Tong University, China approved this study. Written consent was given by the patients for their information to be stored in the hospital database and used for research.

### Patients and data collection

We included all patients aged 18 years or older who commenced PD between January 1, 1995 and June 30, 2012 at Ren Ji Hospital, School of Medicine, Shanghai Jiao Tong University, had received PD treatment for at least 90 days, and were diagnosed with diabetes mellitus at the initiation of PD. Patients were divided into two groups by their primary renal disease—diabetic nephropathy (DN) group and non-diabetic nephropathy (NDN) group. For each enrolled patient, we evaluated their medical records carefully and made the diagnosis based on their medical course, whether biopsy being done, existing of another etiological disease (such as polycystic kidney disease, lupus nephritis, obstructive nephropathy, etc.) and evidence of microvascular diabetic complications, etc.

Demographic data and laboratory parameters were collected at baseline. The baseline was defined as the time when patients received the first clinical evaluation within 3 months after the initiation of PD. The following demographic and comorbidity characteristics were collected: age, gender, underlying cause of ESRD, diabetic duration, height, weight, body mass index (BMI) [BMI = weight (kg) /height (m^2^)], presence of comorbid diseases such as hypertension, cardiovascular disease (CVD), blindness due to diabetic retinopathy, chronic pulmonary disease and malignancy. CVD was defined as a previous history of coronary artery disease, peripheral vascular disease or cerebrovascular disease. The baseline laboratory parameters were collected as follows: fasting plasma glucose, hemoglobin, serum albumin, corrected calcium, phosphate, intact parathyroid hormone (iPTH) and lipids. Baseline dialysis adequacy data included urine output, ultrafiltration volume, residual renal function (RRF), and indices of urea kinetics (Kt/Vurea) and creatinine clearance (CrCl) for both peritoneal and residual renal function. RRF was calculated by the mean of renal clearances of urea and creatinine from a 24-hour urine collection. Peritoneal transport characteristics were measured by the dialysate to plasma creatinine ratio (D/Pcr) at 4 hour in a standard peritoneal equilibration test. Other clinical data were also obtained as follows: blood pressure, PD regimen, dialysate glucose exposure (calculated from the dialysis regimen as described by Davies *et al* [7]), dialysate glucose absorption (total dialysate glucose absorption per day = (1-D4/D0) × total glucose exposure per day, as described by Donna M. Bodnar *et al* [8]), normalized protein nitrogen appearance (nPCR) (from 24-hour dialysate and urine collections), records of taking angiotensin-converting enzyme inhibitors or angiotensin receptor blockers (ACEI/ARBs), steroid therapy and insulin.

During the follow-up, the total number of episodes of peritonitis and the date of first episode of peritonitis were collected. Peritonitis rates were calculated by dividing the months of PD at risk by the number of episodes. We also calculated the RRF decline rate during the first 6 month after PD initiation.

All the patients in the present study used glucose-containing dialysate. Detailed causes for death and transferring to hemodialysis (HD) were recorded. Causes for death were categorized as follows: CVD including cardiac, cerebrovascular, peripheral vascular and sudden death; infection, including peritonitis and other infections; other and unknown causes. Causes for switching to HD were categorized as follows: peritonitis; inadequate dialysis; medical problems such as pleuroperitoneal communication; and patient preference.

All patients were followed up from the date of PD initiation until death, cessation of PD, transferred to other PD centers or to the end of the study (June 30, 2013). All patients were dialyzed with glucose-based dialysis solution (DIANEAL, Baxter).

### Statistical analyses

Summaries of continuous variables were presented as means ± SD for normally distributed data and as medians with interquartile ranges for skewed data; categorical variables were presented as frequencies (percentages). Continuous variables were compared by Student’s t-test for parametric data or the Mann—Whitney test for nonparametric data. Comparisons of categorical data were made by chi-square test or Fisher’s exact test, as appropriate.

The actuarial cumulative survival rates were calculated by life table method. Survival estimates and curves were generated according to the Kaplan—Meier method and compared by the log-rank test. Risk factors predictive of outcomes were first determined by univariate Cox analysis. Variables with P <0.15 in the univariate Cox analysis were presented for multivariable Cox regression analysis, together with age, gender, CVD comorbidity and diabetes as a comorbid or not, RRF and D/Pcr. A backward stepwise elimination procedure was applied and only covariates that remained significant at P<0.05 were kept in the model. Results were expressed at the last step as the hazard ratio (HR) and 95% confidence interval (95% CI).

Statistical analysis was performed using SPSS version 19.0 (SPSS Inc., Chicago, IL, USA). A P-value <0.05 was considered statistically significant.

## Results

### Demographic and clinical characteristics

A total of 1004 patients entered our PD program and started PD between January 1, 1995 and June 30, 2012. Among them 163 patients (16.2% of all patients) were included in the final dataset, including 121 (74.2%) with diabetes as a primary renal disease and 42 (25.8%) with diabetes as a comorbid condition. Patient demographic and clinical data are listed in Table 1. There were 3 patients diagnosed with Type 1 DM, and the rest were diagnosed with Type 2 DM. The underlying renal diagnosis of NDN group was glomerulonephritis in 15 (35.7%), hypertensive nephrosclerosis in 11 (26.2%), polycystic kidney disease in 3 (7.1%), autoimmune diseases in 4 (9.5%), other causes in 6 (14.3%), and unknown causes in 3 patients (7.1%). When compared with DN group, patients in NDN group had a shorter diabetic duration (4.0, 2.0–7.0 vs. 15.0, 10–20.0 year, P < 0.001), had a lower fasting plasma glucose level (6.42, 5.24–7.92 vs. 7.53, 5.60–9.8 mmol/L, P = 0.032), had a higher plasma C-reactive protein (CRP) level (4.53, 1.43–12.71 vs. 3.00, 1.07–4.72 mg/l, P = 0.011). Patients in NDN group were more likely to take steroid [4 (9.5%) vs. 1 (0.8%), p = 0.005] and were less likely to take insulin treatment [24 (57.1%) vs. 108 (89.3%), P < 0.001].

**Table 1:** Demographic and baseline characteristics of the study patients.

Variables	DN group	NDN group	P value
N	121	42	
Age (yr)	61.5 ± 12.8	65.3 ± 12.0	0.094
Gender [male (%)]	73 (60.3%)	21 (50.0%)	0.243
BMI (Kg/m^2^)	22.7 (20.9, 24.9)	23.3 (21.5, 25.6)	0.367
Primary renal disease, n (%)			<0.001
Diabetic nephropathy	121(100%)	0 (0.0%)	
Glomerulonephritis	0	15 (35.7%)	
Hypertension nephrosclerosis	0	11 (26.2%)	
Polycystic kidney disease	0	3 (7.1%)	
Autoimmune diseases	0	4 (9.5%)	
Others causes	0	6 (14.3%)	
Unknown causes	0	3 (7.1%)	
Comorbidity, n (%)			
Hypertension	98 (81.0%)	36 (85.7%)	0.491
CVD	54 (44.6%)	24 (57.1%)	0.162
Blindness due to diabetic retinopathy	8 (6.6%)	0 (0.0%)	0.114
Chronic pulmonary disease	7 (5.8%)	2 (4.8%)	1.000
Malignancy	3 (2.5%)	2 (4.8%)	0.604
Diabetic duration (year)	15.0 (10, 20.0)	4.0 (2.0, 7.0)	<0.001
Blood pressure (mmHg)			
Systolic	148 (135, 160)	140.0 (131, 160)	0.566
Diastolic	80 (71, 90)	82 (71, 90)	0.882
Laboratory measures			
Fasting plasma glucose (mmol/L)	7.53 (5.60, 9.80)	6.42 (5.24, 7.92)	0.032
Hemoglobin (g/l)	105.3 ± 21.0	99.5 ± 24.6	0.137
Serum albumin (g/l)	32.1 ± 5.2	33.5 ± 5.7	0.144
Total cholesterol (mmol/l)	4.80 ± 1.22	5.21 ± 1.38	0.075
Triglyceride (mmol/l)	1.54 (1.09, 2.31)	1.71 (1.28, 2.60)	0.195
Low-density lipoprotein (mmol/l)	2.84 ± 0.87	2.91 ± 0.95	0.679
Phosphate (mmol/l)	1.35 (1.13, 1.66)	1.40 (1.19, 1.75)	0.196
Corrected calcium (mmol/l)	2.28 (2.15, 2.42)	2.32 (2.19, 2.45)	0.415
iPTH (pmol/l)	180.0 (54.2, 322.0)	166.0 (34.1, 387.0)	0.988
CRP (mg/l)	3.00 (1.07, 4.72)	4.53 (1.43, 12.71)	0.011
Use of ACEI/ARB (%)	80 (72.1%)	22 (59.5%)	0.151
Steroid therapy (%)	1 (0.8%)	4 (9.5%)	0.005
Insulin treatment (%)	108 (89.3%)	24 (57.1%)	< 0.001

### PD prescription and adequacy data

At the initiation of PD, NDN group were treated with significantly lower dialysate dose (6.0, 6.0–6.0 vs. 6.0, 6.0–8.0 l/day, P = 0.008). They also had a lower dialysate glucose exposure (110, 90–130 vs. 120, 90–150 g/day, P = 0.023), a lower peritoneal CrCl (34.9 ± 10.2 vs. 38.9 ± 10.5 l/week/1.73 m^2^) and a higher nPCR level (0.86, 0.80–1.02 vs. 0.80, 0.70–0.97 g/kg/d, P = 0.042). NDN patients tended to lose their RRF faster than DN patients during the first 6 months [-0.11 (-0.41, 0.03) vs. -0.31 (-0.64, -0.08) ml•min-1•month-1, P = 0.051] (Table 2).

**Table 2:** PD prescription and adequacy data.

Variables	DN group	NDN group	P value
N	121	42	
Dialysate dose(l/day)	6.0 (6.0, 8.0)	6.0 (6.0, 6.0)	0.008
Dialysate glucose exposure (g/day)	120 (90, 150)	110 (90, 130)	0.023
Dialysate glucose absorption (g/day)	69.9 (53.1, 89.3)	68.1 (49.6, 81.4)	0.174
RRF(ml/min)	3.94 (1.89, 5.48)	3.98 (1.69, 6.23)	0.714
Urine volume (ml/day)	800 (450, 1200)	900 (350, 1575)	0.336
Peritoneal Ultrafiltration (ml/day)	300 ± 539	226 ± 644	0.479
Total Kt/Vurea	2.17 ± 0.58	2.25 ± 0.73	0.535
Peritoneal Kt/Vurea	1.39 ± 0.33	1.30 ± 0.44	0.245
Renal Kt/Vurea	0.80 ± 0.61	0.95 ± 0.81	0.213
Total CrCl (l/week/ 1.73 m^2^)	79.2 ± 29.4	82.9 ± 43.2	0.553
Peritoneal CrCl (l/week/1.73 m^2^)	38.9 ± 10.5	34.9 ± 10.2	0.046
Renal CrCl (l/week/1.73 m^2^)	40.0 ± 30.7	48.0 ± 46.6	0.227
nPCR (g/kg/d)	0.80 (0.70, 0.97)	0.86 (0.80, 1.02)	0.042
D/Pcr	0.68 ± 0.13	0.67 ± 0.15	0.705
RRF decline rate during the first 6 months(ml•min^-1^•month^-1^)	-0.11 (-0.41, 0.03)	-0.31 (-0.64, -0.08)	0.051

### Patient survival and predictors of mortality

**Fig 1:**
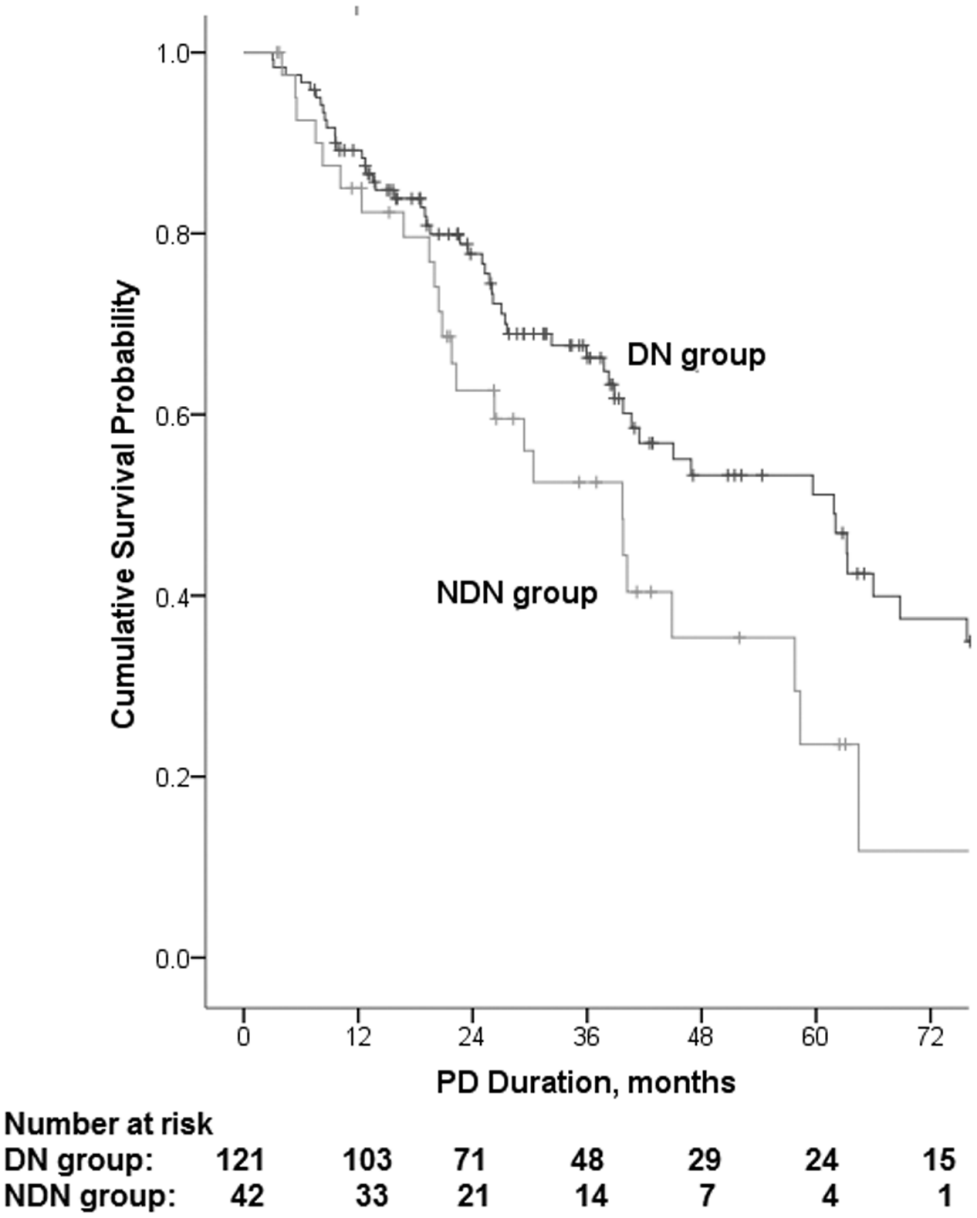
Kaplan—Meier survival analysis for patient survival. Kaplan—Meier analysis showed that patient survival in NDN group was significantly inferior to DN group (log rank 4.830, P = 0.028).

Table 3 shows the data of patients who died, switched to HD, lost to follow-up or were still on PD. With a total follow-up of 5531.1 patient-months, the median duration of follow-up was 28.7 (15.7–47.0) months for DN group and 24.3 (12.35–40.5) months for NDN group. By the end of the study 55(45.5%) patients in DN group died during the follow-up period, due to CVD (n = 27, 49.1%), followed by infection (n = 10, 18.2%) and other and unknown causes (n = 18, 32.7%). Twenty-five (59.5%) patients died in NDN group, due to CVD (n = 12, 48.0%), infection (n = 4, 16.0%) and other and unknown causes (n = 9, 36.0%). The distribution of causes of death was not different between these two groups (P>0.05) (Table 4). Actuarial patient survival rates were 89%, 78%, 66% and 51% for DN group, and 85%, 63%, 53% and 25% for NDN group at 1, 2, 3 and 5 years. Kaplan—Meier survival curves for DN group and NDN group are shown in Fig 1. Patient survival in NDN group was significantly inferior to DN group (log rank 4.830, P = 0.028). Table 5 shows the results of univariate and multivariate Cox analysis of baseline variables in relation to mortality. Multivariate Cox proportional hazards modelling showed that older age (HR 1.047, 95% CI 1.022–1.073, p<0.001) and CVD comorbidity (HR 2.200, 95% CI 0.1.269–3.814, P = 0.005) and diabetes as a comorbidity condition (HR 1.806, 95% CI 1.003–3.158, P = 0.038) were the independent predictors of increased mortality in the combined cohort.

**Table 3:** Outcomes of DN group and NDN group.

Outcomes	DN group (n = 121)	NDN group (n = 42)
Death	55 (45.5%)	25 (59.5%)
Switch to HD	15 (12.4%)	4 (9.5%)
Transfer to other centers	10 (8.3%)	4 (9.6%)
Lost to follow-up	1 (0.8%)	0 (0.0%)
Still on PD	40 (33.1%)	9 (21.4%)

**Table 4:** Causes of death in DN group and NDN group.

Causes	DN group (n = 55)	NDN group (n = 25)
CVD	27 (49.1%)	12 (48.0%)
Peritonitis	5 (9.1%)	2 (8.0%)
Other infections	5 (9.1%)	2 (8.0%)
Other and unknown	18 (32.7%)	9 (36.0%)

**Table 5:** Results of univariate and multivariate Cox analysis of patient survival in all diabetic peritoneal dialysis (PD) patients.

Characteristic	Univariate		Multivariate	
	HR (95% CI)	*P* value	HR (95% CI)	*P* value
Age	1.056 (1.035, 1.079)	< 0.001	1.047 (1.022, 1.073)	< 0.001
CVD	2.464 (1.548, 3.922)	< 0.001	2.200 (1.269, 3.814)	0.005
Group factor	1.701 (1.053, 2.743)	0.030	1.806 (1.003, 3.158)	0.038
Gender	0.697 (0.446, 1.089)	0.113		0.894
BMI	1.024 (0.958, 1.093)	0.489		0.646
Serum albumin	0.937 (0.897, 0.979)	0.004		0.072
Hemoglobin	0.985 (0.974, 0.997)	0.011		0.185
Residual renal function	0.987 (0.917, 1.062)	0.730		0.223
D/P_cr_	1.311 (0.201, 8.557)	0.777		0.564

### Technique Survival

**Fig 2:**
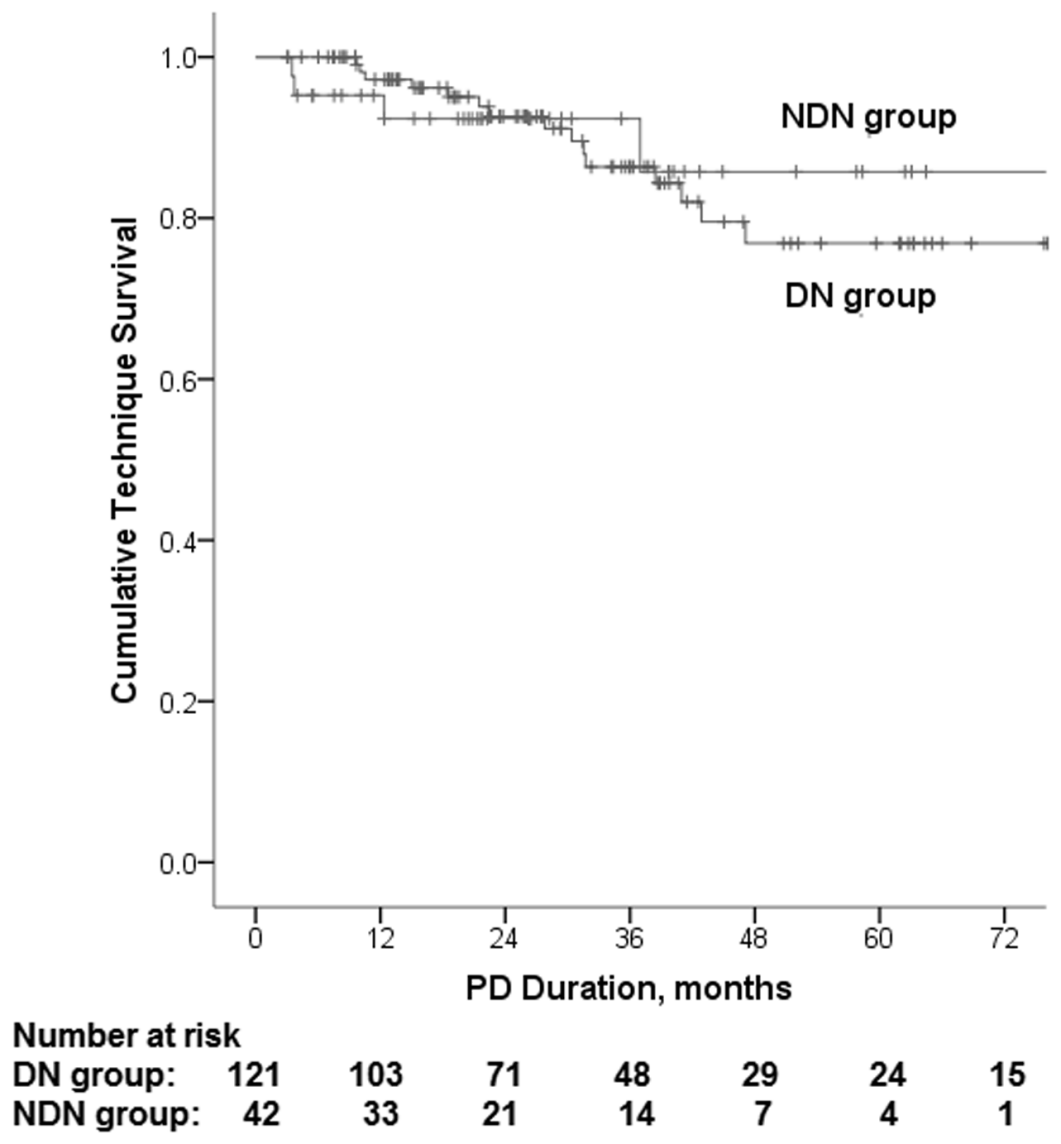
Kaplan—Meier survival analysis for technique survival. Kaplan—Meier analysis showed that technique survival was not significantly different between DN group and NDN group (log rank 0.040, P = 0.841).

By the end of the study 15 (12.4%) patients had been transferred to HD in DN group, due to peritonitis (n = 12), medical problem (n = 1), inadequate dialysis (n = 1), and patient preference (n = 1). For NDN group, 5 (10.2%) patients had been transferred to HD, due to peritonitis (n = 3) and medical problem (n = 1) (Table 6). The 1-, 2-, 3- and 5 year technique survival rates were 97%, 93%, 87% and 78% for DN group, and 95%, 91%, 91% and 83% for NDN group. Kaplan—Meier analysis showed that technique survival was not significantly different between the two groups (log rank 0.040, P = 0.841) (Fig 2).

**Table 6:** Causes of transferring to HD in DN group and NDN group.

Causes	DN group (n = 15)	NDN group (n = 4)
Peritonitis	12 (80.0%)	3 (75.0%)
Medical problem	1 (6.7%)	1 (25.0%)
Inadequate dialysis	1 (6.7%)	0 (0.0%)
Patient preference	1 (6.7%)	0 (0.0%)

### Peritonitis Rate and predictors of peritonitis-free survival

**Fig 3:**
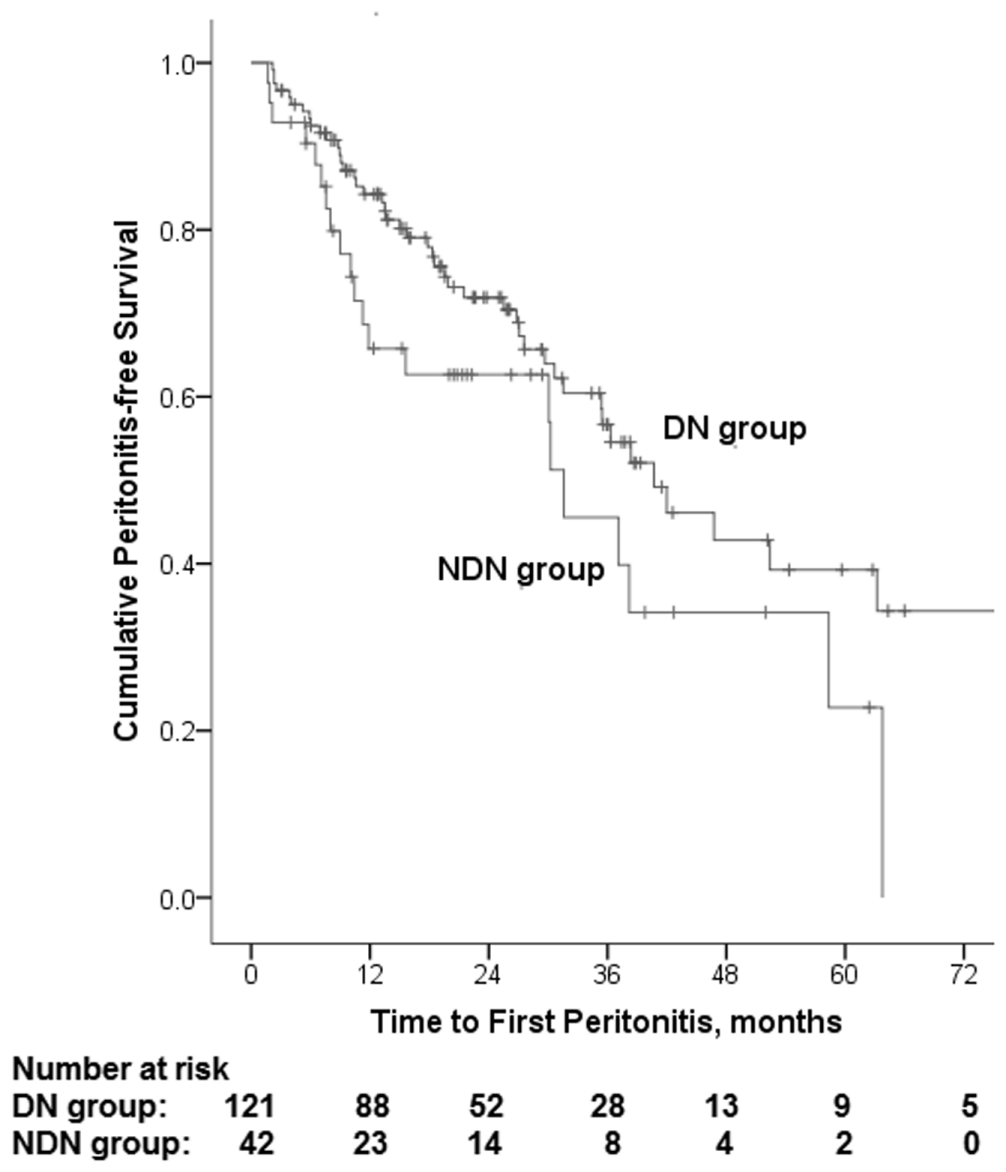
Kaplan—Meier survival analysis for time to first peritonitis episode. Kaplan—Meier analysis showed that NDN group had a marginally worse peritonitis-free survival compared with DN group (log rank 3.297, P = 0.069).

During the study period, a total of 115 episodes of peritonitis occurred, including 79 in DN group and 36 in NDN group. The average peritonitis rate was one episode every 54.5 patient months in DN group and one episode every 36.1 patient months in NDN group. The median peritonitis-free period for DN group was 40.7 months and 31.6 months for NDN group respectively. The 1-, 2-, 3- and 5 year peritonitis-free survival rates were 84%, 72%, 57% and 41% for DN group, and 67%, 63%, 48% and 24% for NDN group. Kaplan—Meier analysis showed NDN group had a marginally worse peritonitis-free survival than that of DN group (log rank 3.297, P = 0.069) (Fig 3). Table 7 shows the results of univariate and multivariate Cox analysis of peritonitis-free survival in all diabetic PD patients. Multivariate cox survival analysis showed that higher CRP level was the predictor of shorter duration to first peritonitis episode (HR 1.025, 95% CI 1.011 to 1.040, P = 0.001) for overall population.

**Table 7:** Results of univariate and multivariate Cox analysis of peritonitis-free survival in all diabetic peritoneal dialysis (PD) patients.

Characteristic	Univariate		Multivariate	
	HR (95% CI)	*P* value	HR (95% CI)	*P* value
Age	1.010 (0.989, 1.013)	0.363		0.926
Gender	1.135 (0.694, 1.857)	0.614		0.862
BMI	0.998 (0.928, 1.073)	0.956		0.783
CVD	0.760 (0.451, 1.279)	0.302		0.202
Group factor	1.670 (0.992, 2.812)	0.054		0.070
Serum albumin	1.015 (0.968, 1.064)	0.543		0.073
Serum CRP	1.026 (1.011, 1.040)	< 0.001	1.025 (1.011, 1.040)	0.001
Total Kt/V_urea_	0.680 (0.424, 1.090)	0.109		0.149
Steroid therapy	3.555 (1.272, 9.934)	0.016		0.077

## Discussion

In our study, we found that PD patients with diabetes as a comorbidity had an inferior patient survival compared with those with diabetes as a primary renal disease. PD patients with diabetes as a comorbidity had a marginally shorter peritonitis-free period, however, technique survival was not significantly different between the two groups.

Patients with diabetes as a comorbid condition accounted for 25.8% of the overall diabetic PD patients in our study cohort. This proportion was similar to that from other reports [3, 6, 9]. At PD initiation, patients in NDN group had a lower fasting plasma glucose level than those in DN group. A previous study found that lower glucose exposure may decrease the metabolic disorders including hyperglycemia, hyperinsulinemia and insulin resistance [10]. Since patients in NDN group received significantly less dialysate glucose exposure, it might be one of the reasons for their lower initial Fasting plasma glucose level. We noted that plasma CRP level was higher in NDN group, indicating that these patients may have more severe inflammatory states. We did not know the exact causes for this finding. Since elevated plasma CRP level was closely related to older age [11] and CVD comorbidity [12], the possible explanation could be that NDN group tended to be older and to have a higher CVD prevalence. However, neither of the parameters was reaching statistical significance.

Even though PD patients with diabetes as a comorbidity accounted for a certain proportion, there were only few studies investigating their outcomes. Schroijen MA et al found that PD patients with diabetes a comorbidity had a similar mortality to PD patients without diabetes, while PD patients with diabetic nephropathy had a worse mortality than those without diabetes, indicating that PD patients with diabetes as a comorbidity had a better patient survival than those with diabetic nephropathy [4]. Recently, a large-sample study reported that among younger dialysis patients (age < 70 years), those with diabetic nephropathy had a 1.28-time increased mortality than those with diabetes as a comorbidity. While among dialysis patients older than 70 years, these two groups had a similar patient survival [5]. Another study included 18 diabetic dialysis patients with polycystic kidney disease as their etiology and 18 dialysis patients with diabetic nephropathy, and found that these two groups had a similar patient survival [6]. Theoretically, PD patients with diabetic nephropathy may have a worse outcome than those with diabetes as a comorbidity because of their possibly longer diabetic course and thus more severe multi-organ complications. However we found in present study that PD patients with diabetes as a comorbidity had a worse patient survival than those with diabetic nephropathy. There were several possible explanations contributing to our findings. Firstly, the higher mortality in NDN group may due to their more severe inflammation status indicated by elevated plasma CRP. There were quite a few studies that showed increased plasma CRP level was associated with mortality or cardiovascular events in PD patients. Ducloux D et al found that among PD patients, elevated CRP level (plasma CRP>3.2 mg/L) raised the cardiovascular event by 2.34–5.41 times and raised the mortality by 1.41–5.2 times [13]. Wang AY et al reported that every 1 mg/L increase in hs-CRP was independently predictive of higher all-cause mortality (HR 1.02) and cardiovascular mortality (HR 1.03) in PD patients [14]. Another prospective cohort study of 50 PD patients with 3-year follow-up after the initial determination of CRP and showed that an elevated plasma CRP (>6.0 mg/L) was predictive of myocardial infarction (HR 4.8) [15]. Additionally, it has been reported that increased CRP level was associated with factors that played important roles in PD patient survival, such as hypoalbuminemia [16], residual renal function [17], and peritoneal transport rate [18]. Therefore, it was not surprising that patients in NDN group, who had a significantly higher plasma CRP level at PD initiation, showed a worse patient survival. Secondly, patients in NDN group may have a higher risk of new onset of CVD events than those in DN group. Secondly, it has been reported that having diabetes as a comorbidity rather than a primary diagnosis predicts earlier loss of RRF [19] and fast declining RRF is associated closely with mortality in PD patients [20]. The NDN patients in present study tended to lose their RRF faster than DN patients might also be one of the explanations that NDN patients had higher mortality than DN patients. Recently a retrospective large-sample study analyzed the risk of new onset CVD events among diabetic ESRD patients (diabetes before ESRD) and de novo diabetic ESRD patients (diabetes after ESRD), after stratified by age. The results showed that in all ages except for those aged between 40 and 49 years, de novo diabetic ESRD patients had a higher risk of new onset acute myocardial infarction than diabetic ESRD patients. Besides, among those aged between 60 and 79, de novo diabetic ESRD patients had a higher risk of stroke than diabetic ESRD patients. This study indicated that de novo diabetes, of which the diabetic course may be shorter, had a greater burden on acute myocardial infarction and stroke compared to diabetes before ESRD [21]. The NDN patients in present study were much more similar to those do novo diabetic ESRD patients, since both of them had non-diabetic etiology and had a shorter diabetic course than those with diabetic nephropathy. Therefore, new onset of acute myocardial infarction and stroke might also be one of the reasons why NDN group had a higher mortality. The mechanism might be that diabetes mellitus and non-diabetic renal disease shared some similar pathophysiological mechanisms leading to CVD events. However, some other mechanisms may be unique and work synergistically to accelerate the occurrence of CVD events [21]. Clearly more studies are needed to confirm the hypothesis.

We also found that patients in NDN group had a marginally shorter peritonitis-free period than those in DN group and CRP was an independent risk for peritonitis-free survival after adjustment. It has been reported that a progressive serum hs-CRP within the first year predicted the first two-year peritonitis risk in PD patients, indicating serum CRP may act as a predictor for short-term peritonitis [22].

Our study had several limitations. Firstly, this was a single-center retrospective study. The sample size is relatively small and it might fail to detect a statistical significance in some characteristics between these two groups. However we had detailed data and avoided the potentially confounding influence of treatment practice varying by PD programs. Secondly, we did not collect HbA1C or glycated albumin level, which were thought to be superior indicators for monitoring glycemic control. Thirdly, not all patients had renal biopsy to determine their diagnosis of renal disease. However, as for all the subjects with no renal biopsy, the diagnosis of primary renal disease was reviewed and made carefully according to their clinical features.

In conclusion, PD patients with diabetes as a comorbidity had an inferior patient survival compared to those with diabetic nephropathy, and closer monitoring and extra attention in the former subgroup of patients are therefore warranted.
